# Quantification of Gaze-Dependent Corrective Errors With Fresnel Prisms: Implications for Strabismus Management

**DOI:** 10.1167/iovs.67.5.68

**Published:** 2026-05-27

**Authors:** Eizo Tanaka, Shin Morisawa, Yuki Morizane, Tomoki Tokutake, Satoshi Hasebe

**Affiliations:** 1 Department of Ophthalmology, Graduate School of Medicine, Dentistry and Pharmaceutical Sciences, Okayama University, Okayama, Japan; 2 Department of Ophthalmology II, Kawasaki Medical School, Okayama, Japan

**Keywords:** Fresnel prisms, corrective errors, prism adaptation test, strabismus, binocular vision

## Abstract

**Purpose:**

Fresnel prisms are widely used to manage strabismus. Although prism power is typically selected based on the deviation in the primary gaze position, actual deflection varies with the gaze direction because the angle of incidence changes. This study quantified gaze-dependent corrective errors in the secondary and tertiary positions.

**Methods:**

Fresnel prisms (10Δ–40Δ) were mounted on a two-axis rotation stage. Deflection was measured by projecting a laser spot onto a tangent screen 100 cm away while rotating the prisms horizontally and/or vertically (±30°). Two configurations were tested: eyeward prism serration (EPS) and outward prism serration (OPS). Geometric modeling was used to predict the deflection of a prism attached to a spectacle lens, and corrective errors across the gaze field were visualized as contour maps using Kriging interpolation.

**Results:**

Corrective errors varied with prism power, gaze position, and application method (EPS vs. OPS; factorial ANOVA, *P* < 0.05). Prisms > 20Δ exhibited substantial, spatially complex errors. In the EPS configuration, horizontal gaze shifts toward the apex caused rapidly increasing overcorrection, whereas shifts toward the base led to undercorrection. In the OPS configuration, this gradient was reversed on the horizontal axis (*P* < 0.0001). Vertical gaze shifts not only amplified the horizontal corrective effects but also created unintended vertical components (perpendicular to the principal section).

**Conclusions:**

Fresnel prisms introduce nonlinear, gaze-dependent corrective errors despite the full correction of the strabismic deviation in the primary position. Recognition of these optical properties may help optimize therapeutic outcomes and enhance diagnostic accuracy.

Fresnel membrane prisms are thin, flexible sheets composed of miniature prism elements that can be attached to spectacle lenses to correct strabismic deviations of up to 40Δ per eye. They are widely utilized in the management of strabismus and are particularly effective for acute or variable conditions, such as fourth and sixth cranial nerve palsies, thyroid eye disease, posttraumatic diplopia, convergence or divergence insufficiency, and diplopia following cataract surgery.^1^^,^^2^ They are also employed in the prism adaptation test (PAT).^2^^–^^9^ In this procedure, progressively stronger prisms are applied to neutralize the deviation, and ocular alignment is monitored over minutes to several days until the deviation stabilizes. The goal of PAT is to unmask the maximum deviation that would otherwise be compensated for by slow fusional vergence and thereby reduce the risk of surgical undercorrection. Nevertheless, despite decades of clinical use, its efficacy remains controversial.^2^^–^^12^

Fresnel prism power is typically prescribed according to the angle of strabismus measured in the primary gaze position. However, because prism deflection varies with the angle of incidence, as predicted by Snell's law, corrective errors inevitably arise once gaze moves away from the primary position. This issue is conceptually the same as the findings of Thompson and Guyton,^13^ who showed that the measured angle of strabismus with block prisms varies depending on prism positioning (e.g., frontal-plane, minimum-deviation, or Prentice positions) due to differences in the incident angle.

To the best of our knowledge, the gaze-dependent corrective errors associated with Fresnel prisms have not been previously reported. The present study experimentally and theoretically quantified the magnitude and spatial distribution of these errors in the secondary and tertiary gaze positions. Our findings provide clinically relevant insights that may help optimize therapeutic outcomes and improve diagnostic accuracy.

## Methods

### Bench-Top Experiments

**Figure 1.**
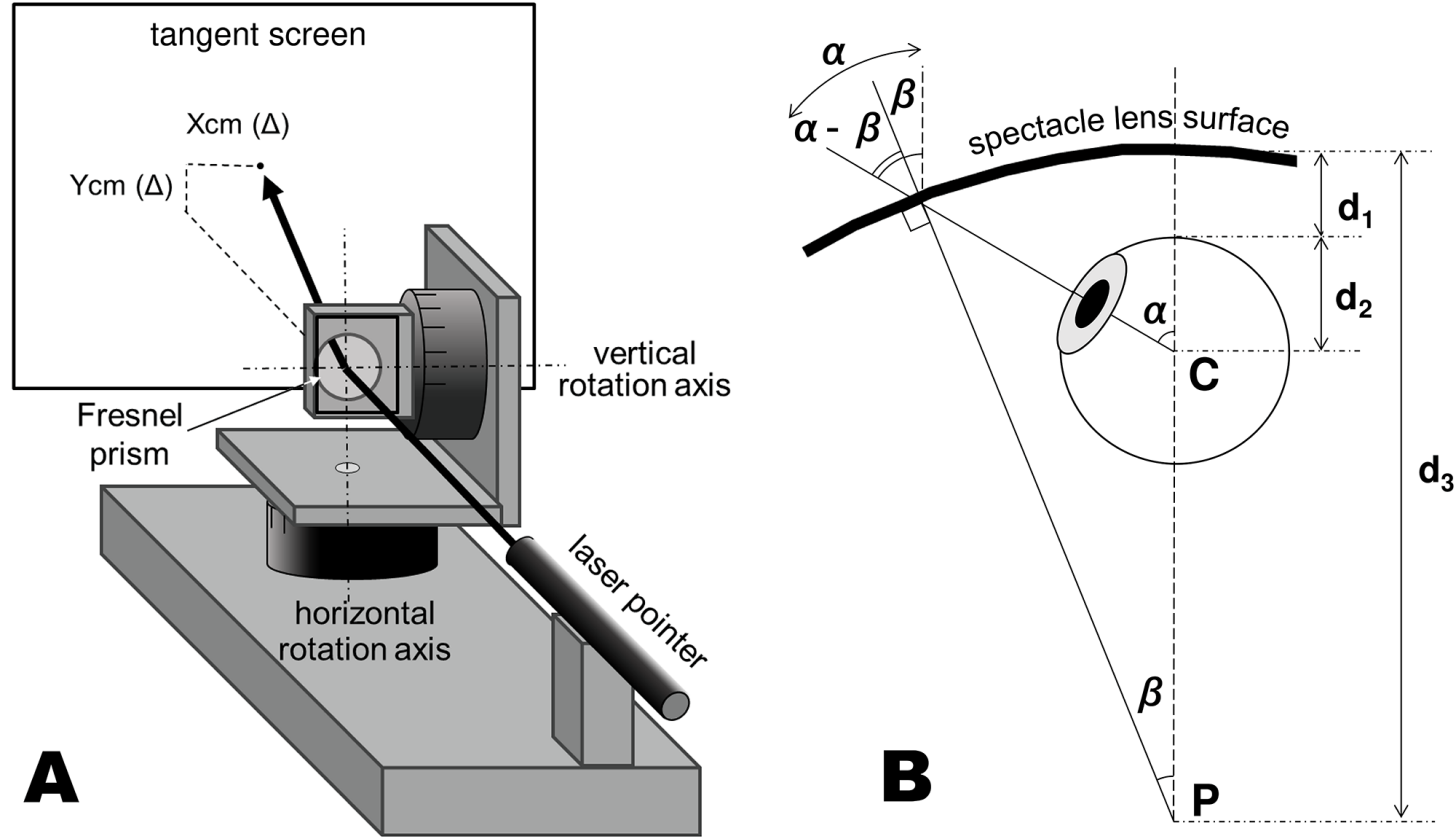
**(**A**, **B**) Schematic illustrations of the experimental set-up (**A**) and the geometric model used to estimate the angle of incidence at the lens surface (**B**).** α, β, *C*, *P*, *d_1_*, *d_2_*, and *d_3_* represent the gaze angle, the tilt angle of the spectacle lens surface, the rotation center of the eye, the center of curvature of the lens base curve, the vertex distance, the distance between the corneal apex and rotation center, and the radius of the lens base curve, respectively. The angle of incidence at the lens surface is indicated by the *double line*.

A Press-On Fresnel prism (10, 20, 30, or 40Δ; 3M, Maplewood, MN, USA) was mounted horizontally (i.e., with the prism serrations aligned to the vertical rotation axis) on a two-axis precision rotation stage (Melles Griot; IDEX Health and Science, Northbrook, IL, USA). To measure prism deflection, we used a laser pointer (ELP-G10; 532-nm wavelength, 1-mW maximum power; KOKUYO, Osaka, Japan) because green light lies near the center of the visible spectrum. Before data collection, the laser pointer, rotation stage, and tangent screen were precisely aligned and positioned on an optical bench (Fig. 1A). A sheet of millimeter-grid paper was affixed to the screen. To ensure its proper orientation, we rotated a 40Δ prism horizontally and verified that the laser trace aligned with the *x*-axis, thereby confirming the proper alignment of the setup.

Measurements were performed in two configurations: eyeward prism serration (EPS), the conventional method, and outward prism serration (OPS), in consideration of the different angles of incidence inherent to each configuration. The examiner wore laser protective glasses (DBY; NoIR, Milford, MI, USA) and marked the location of the laser spot sequentially on the grid paper while rotating the prism horizontally and/or vertically (0°, ±10°, ±20°, or ±30°). Horizontal and vertical deviations of the spot from the origin (spot position without a prism), expressed in centimeters, were taken as the prism deflections (Δ). Prism rotation angles were confirmed using the vernier scale of the rotation stage (precision, 0.1°).

### Statistical Analysis

In the present study, corrective errors were defined as the differences between the observed deflection and the labeled (nominal) prism value. A factorial ANOVA was used to examine the effects of horizontal rotation, vertical rotation, application method (EPS vs. OPS), and prism power. Prism-power effects were summarized using three planned adjacent contrasts (20Δ vs. 10Δ, 30Δ vs. 20Δ, and 40Δ vs. 30Δ), and all interactions were tested within the same model. Assumptions of normality and the homogeneity of variance were confirmed. Model fit was assessed by the global *F* test and adjusted *R*^2^. Parameter estimates with *P* values were calculated for each factor and interaction term. Main and interaction effects were tested via the type III sums of squares. All tests were two tailed. Analyses were performed using JMP 5.01a (SAS Institute, Cary, NC, USA).

### Two-Dimensional Mapping of Corrective Errors Across the Gaze Field

Based on these experimental results, we estimated the corrective errors of the Fresnel prism attached to the spectacle lens using a geometric model (Fig. 1B). Because spectacle lenses have a curved surface, the attached prism gradually tilts outward from the center toward the periphery, which reduces corrective errors in specific gaze directions.^14^ The incidence angle at the lens surface for a given gaze angle α is obtained by subtracting tilt angle β from α. According to this model, the incidence angle may be expressed as a function of α alone as follows:

(1) $$\alpha \;-\;\beta \;=\;\arcsin\;\left[\left(\frac{d_{3}\;-\;d_{1}\;-\;d_{2}}{d_{3}}\right)\;\sin\;\alpha \right]\;.$$

Here, *d*_1_, *d*_2_, and *d*_3_ represent the vertex distance (12 mm), the distance between the corneal apex and the rotation center of the eye (15.3 mm horizontally or 12.5 mm vertically),^15^ and the radius of curvature of the spectacle lens (86 mm), respectively. We then assessed the corrective power at gaze angle α using the experimentally derived relationship between rotation angles (equivalent to the incidence angle at the reference plane) and deflection angles. Corrective errors, defined as the difference between the estimated prism power and the labeled value, were interpolated across gaze positions and visualized as contour maps using Kriging (i.e., Gaussian process regression)^16^^,^^17^ in Surfer (Golden Software, Golden, CO, USA).

## Results

**Figure 2.**
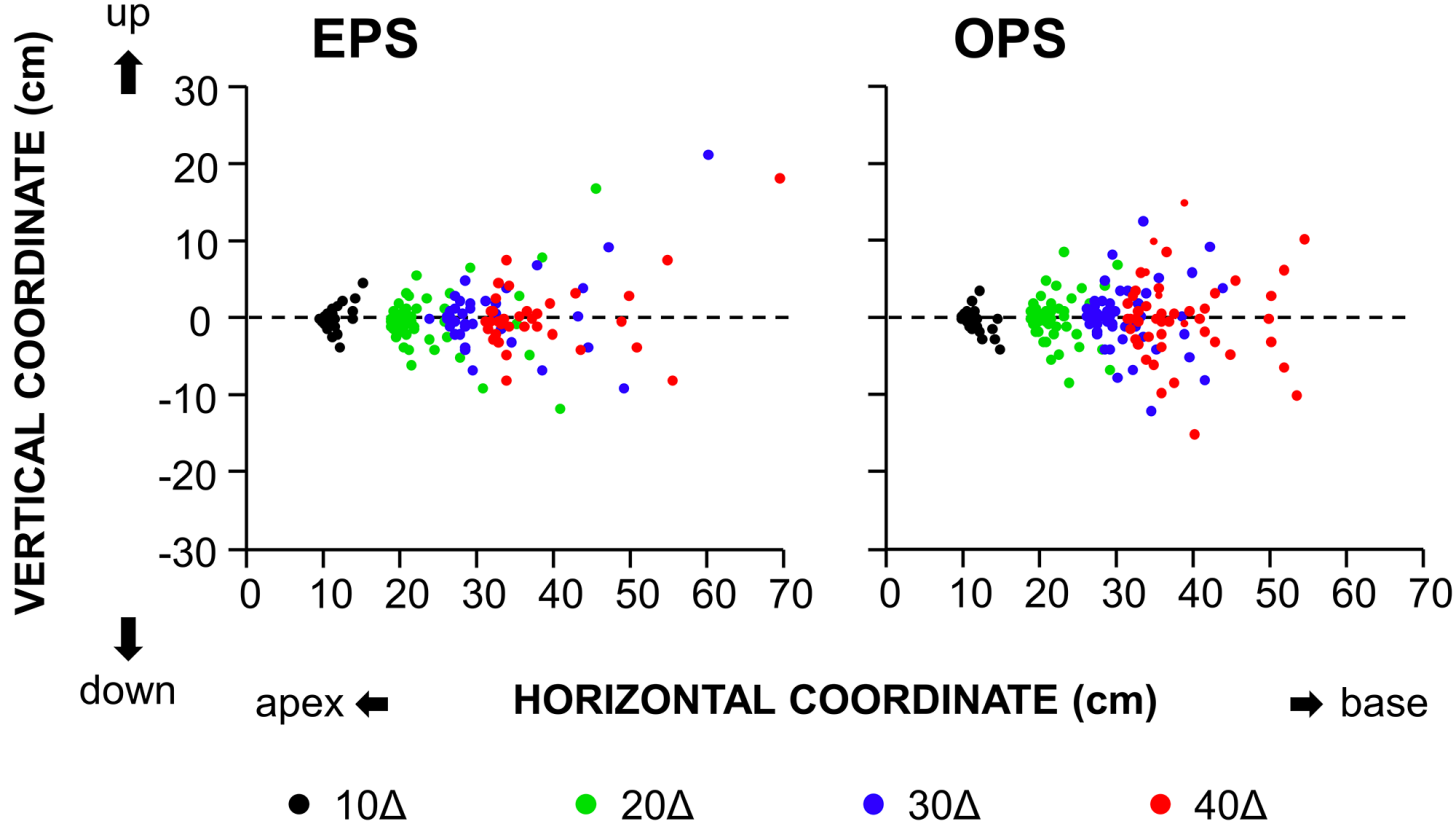
**Source data. All measured laser-spot *x*, *y* coordinates (cm) obtained during horizontal and vertical rotations (±30°) of 10Δ to 40Δ Fresnel prisms in the EPS (*left panel*) and OPS (*right panel*) configurations.** In the EPS configuration, 24 data points could not be recorded because the laser spot became undetectable.

Forty-nine measurements were attempted for each prism and configuration; however, when 30Δ and 40Δ prisms were tested in the EPS configuration, the laser spot became undetectable at some positions (8 and 16 positions, respectively). Figure 2 shows source coordinate data, illustrating clusters of measured laser-spot coordinates around the locations expected from the labeled prism values. As prism power increased, these clusters broadened in both the horizontal and vertical directions. During vertical rotation at a fixed horizontal angle, the spot typically traced a quasi-parabolic trajectory, symmetric about the *x*-axis and opening toward the base. Thus, vertical rotation introduced an unanticipated vertical component (perpendicular to the principal section of the prism) in addition to enhancing horizontal deflection.

Factorial ANOVA indicated that horizontal errors were significantly affected by labeled prism value, configuration, and horizontal rotation (all *P* < 0.05). Two interaction effects were also significant: vertical rotation × horizontal rotation (*P* < 0.01), indicating that the effects of horizontal rotation varied across the levels of vertical rotation, and horizontal rotation × configuration (*P* < 0.0001), indicating that the effect of horizontal rotation differed between EPS and OPS configurations. For vertical errors, the model likewise identified the significant main effects of the labeled value (*P* < 0.0001) and horizontal rotation (*P* < 0.0001), whereas no other main effects or interactions reached significance.

### Horizontal Deflection Changes Induced by Horizontal Prism Rotation

**Figure 3.**
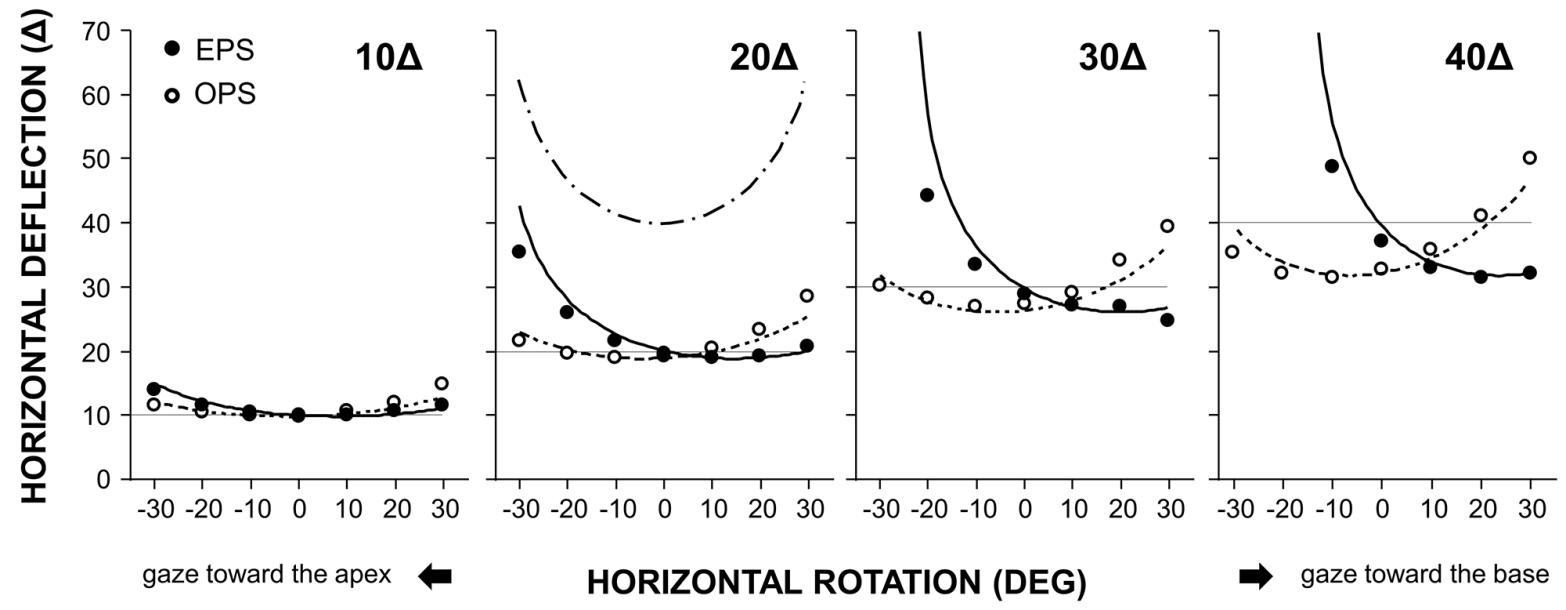
**Horizontal deflection (Δ) as a function of horizontal prism rotation (°) in the EPS and OPS configurations.** Positive and negative horizontal prism rotations correspond to the patient's gaze toward the base and toward the apex, respectively. *Filled* and *open circles* denote the EPS (conventional method) and OPS configurations, respectively. *Solid* and *dashed curves* indicate theoretical values calculated using Snell's law (wavelength, 532 nm; refractive index of polyvinyl chloride, 1.55). The *dash*–*dot curve* indicates the predicted deflection for the EPS configuration with two 20Δ prisms applied symmetrically to both eyes.

Figure 3 shows the relationship between horizontal rotation and horizontal deflection (i.e., along the principal section). The deflection followed a concave-down trajectory, and the gradient increased with prism power. The minimum deflection occurred with gaze toward the base in the EPS configuration and toward the apex in the OPS configuration.

### Validation of Labeled Prism Values

To identify the effective deflection corresponding to the labeled values of commercially available Fresnel prisms, we developed a lookup table (Table). Values that were not directly measured were estimated by fitting cubic regression functions to the measured data. Although the nominal specifications were accurate in the EPS configuration, the OPS configuration showed notable deviations at prism powers > 30Δ, indicating the need for calibration. However, at lower prism powers, the EPS and OPS configurations appeared to be interchangeable for correction in the primary position. Because Fresnel prisms tilt along the lens curvature, their angle of incidence varies with the magnitude and direction of the individual strabismic deviation. Clinically, the final alignment can be confirmed with the alternate cover test.

**Table.** Labeled Prism Values and Corresponding Effective Horizontal Deflections During Horizontal Prism Rotation

	Prism Rotation (°)						
Labeled Value	−30	−20	−10	**0**	+10	+20	+30
Effective deflection (Δ), EPS configuration							
10	14	12	11	10	10	11	12
12	—	14	13	12	12	13	15
15	—	18	17	15	15	15	18
20	35	26	22	20	19	19	21
25	—	34	27	24	23	23	23
30	—	44	33	28	27	26	24
35	—	54	40	33	30	29	27
40	—	64	49	37	33	32	32
Effective deflection (Δ), OPS configuration							
10	12	11	10	10	11	12	15
12	14	12	12	12	13	14	21
15	17	15	15	15	16	18	27
20	22	20	19	19	21	23	34
25	26	24	23	23	25	29	37
30	30	28	26	27	29	34	39
35	33	31	29	30	32	38	42
40	36	32	32	33	36	41	50

Positive and negative rotations correspond to gaze toward the base and toward the apex, respectively. At −30° (EPS), only two data points were available, precluding regression analysis. Values < 10Δ were omitted because the associated errors were considered clinically negligible.

### Simultaneous Horizontal and Vertical Prism Rotations

**Figure 4.**
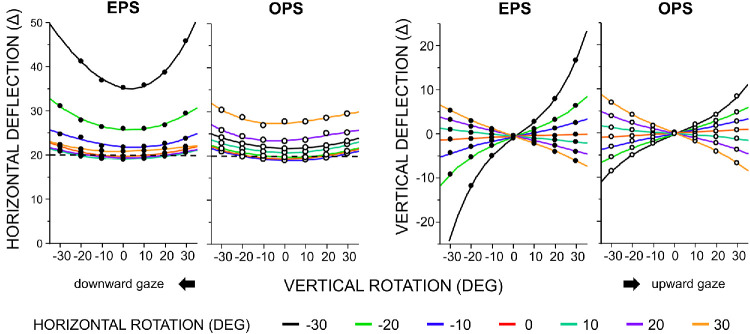
**Horizontal and vertical deflection as functions of vertical prism rotation in the EPS and OPS configurations (20Δ Fresnel prism shown as an example).** Positive and negative values of vertical prism rotation correspond to the patient's upward and downward gaze, respectively. Positive and negative vertical deflections indicate base–down and base–up deflections, respectively. Data were interpolated using cubic polynomial functions. The *dashed line* indicates the labeled prism value.

Under simultaneous horizontal and vertical rotations, the overall response of the horizontal deflection was preserved, whereas its magnitude increased with vertical rotation, particularly in the EPS configuration (left two panels of Fig. 4). Dual-axis rotation also produced a vertical deflection component (i.e., perpendicular to the principal section), as shown in the right two panels of Figure 4. This component increased with vertical rotation and reversed polarity (base-down vs. base-up deflection) depending on the rotation direction. Similar patterns were observed for other prism powers (10Δ, 30Δ, and 40Δ), with the absolute magnitudes of these corrective errors increasing with prism power.

### Corrective Errors of Fresnel Prisms Affixed to a Curved Spectacle Lens Across the Gaze Field

**Figure 5.**
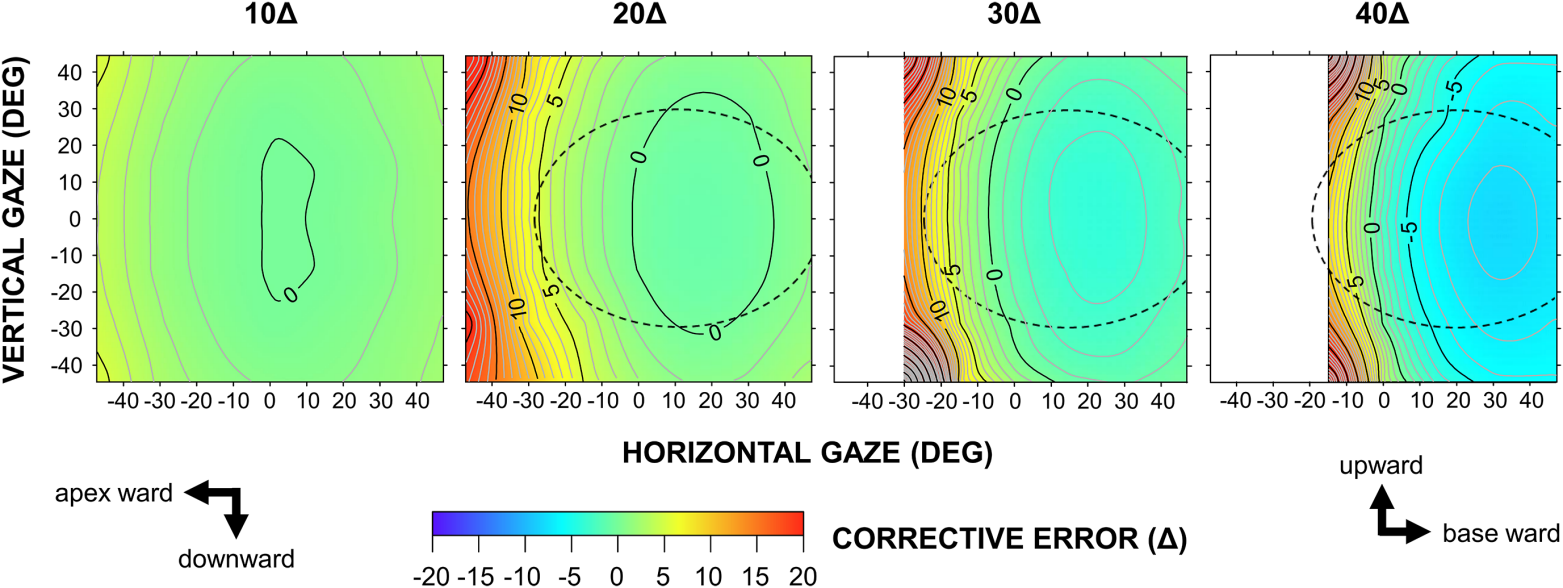
**Horizontal corrective errors along the principal section for 10Δ to 40Δ Fresnel prisms in the EPS configuration.** The maps are centered on the primary gaze position. Positive and negative values indicate overcorrection and undercorrection, respectively. *Contour lines* are plotted at 1Δ intervals. *Dashed lines* outline the size and position of a typical spectacle lens (44 × 31 mm). Blank square areas indicate gaze directions where measurements could not be obtained because the laser spot was undetectable.

Horizontal corrective errors across the gaze field are presented as contour maps (Fig. 5). After correcting for lens curvature using Equation 1, the evaluable field spanned ±47° horizontally and ±44° vertically. In the EPS configuration, errors remained < 5Δ for the 10Δ and 20Δ prisms within the typical spectacle lens area. In contrast, for prism powers > 20Δ, errors increased markedly and showed pronounced horizontal asymmetry: Overcorrection surged sharply with gaze toward the apex, whereas undercorrection increased more gradually toward the base. As shown in Figure 4, vertical gaze shifts amplified horizontal deflection, thereby exacerbating overcorrection or mitigating undercorrection.

**Figure 6.**
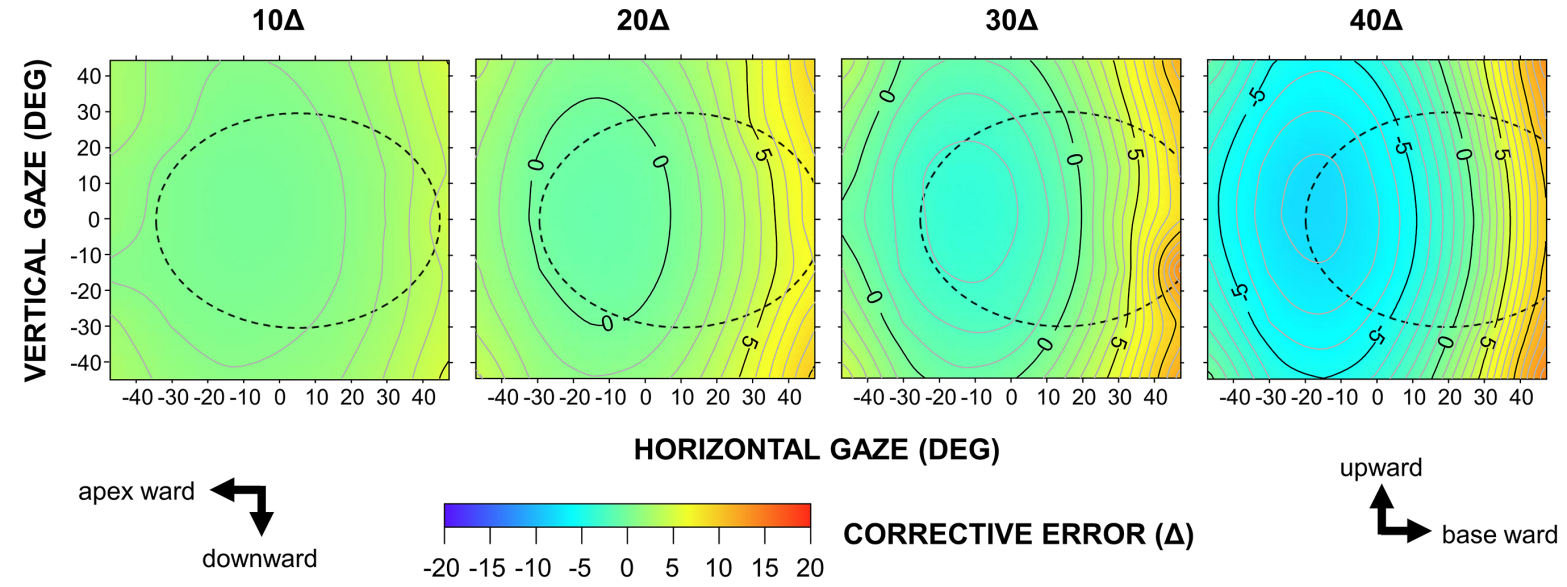
Horizontal corrective errors for 10Δ to 40Δ Fresnel prisms in the OPS configuration.

In the OPS configuration, the horizontal error gradient was reversed (Fig. 6). Gaze toward the apex resulted in increased undercorrection, whereas gaze shifts toward the base led to increased overcorrection. Although these gradients became steeper with increasing prism power, they remained generally shallower than those observed in the EPS configuration. Notably, the zero-error region shifted toward the base as prism power increased, reflecting a growing discrepancy between the labeled value and actual deflection in the primary gaze position.

**Figure 7.**
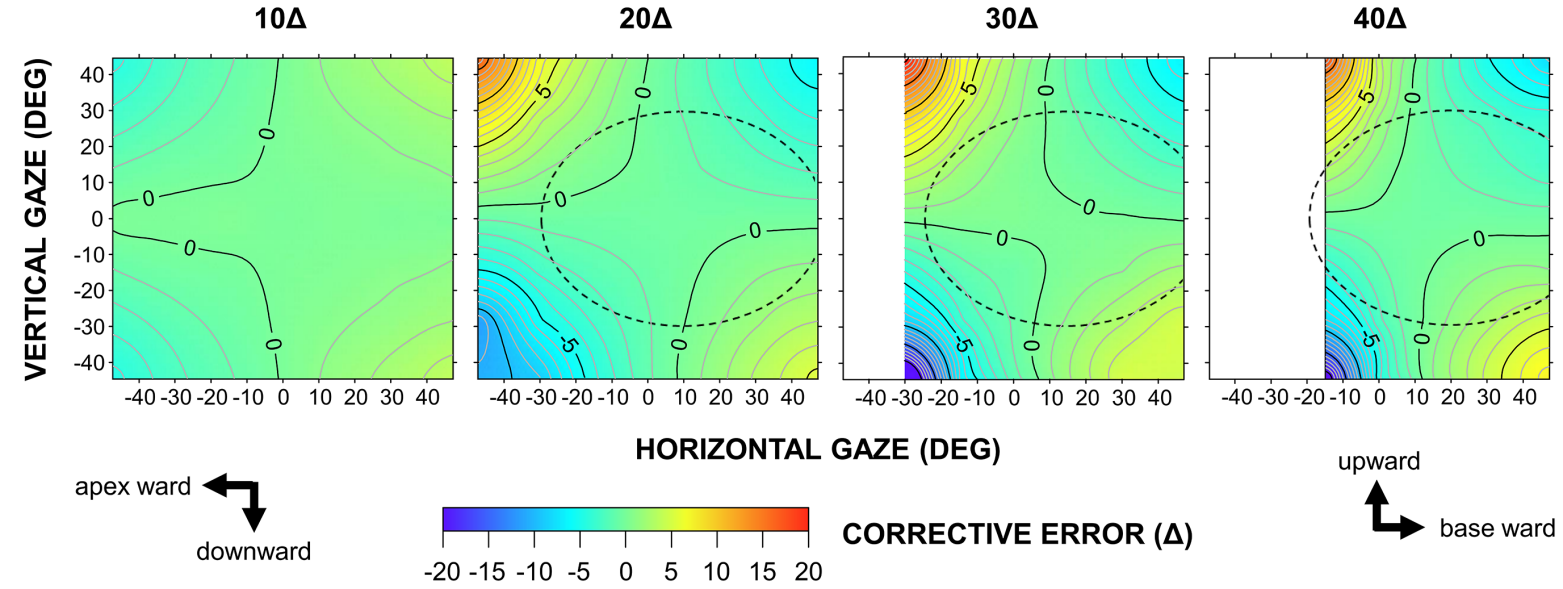
**Vertical corrective errors, measured perpendicular to the principal section, for 10Δ to 40Δ Fresnel prisms in the EPS configuration.** The maps are centered on the primary gaze position. Positive and negative corrective-error values denote base–down and base–up deflections, respectively. *Contour lines* are plotted at 1Δ intervals.

**Figure 8.**
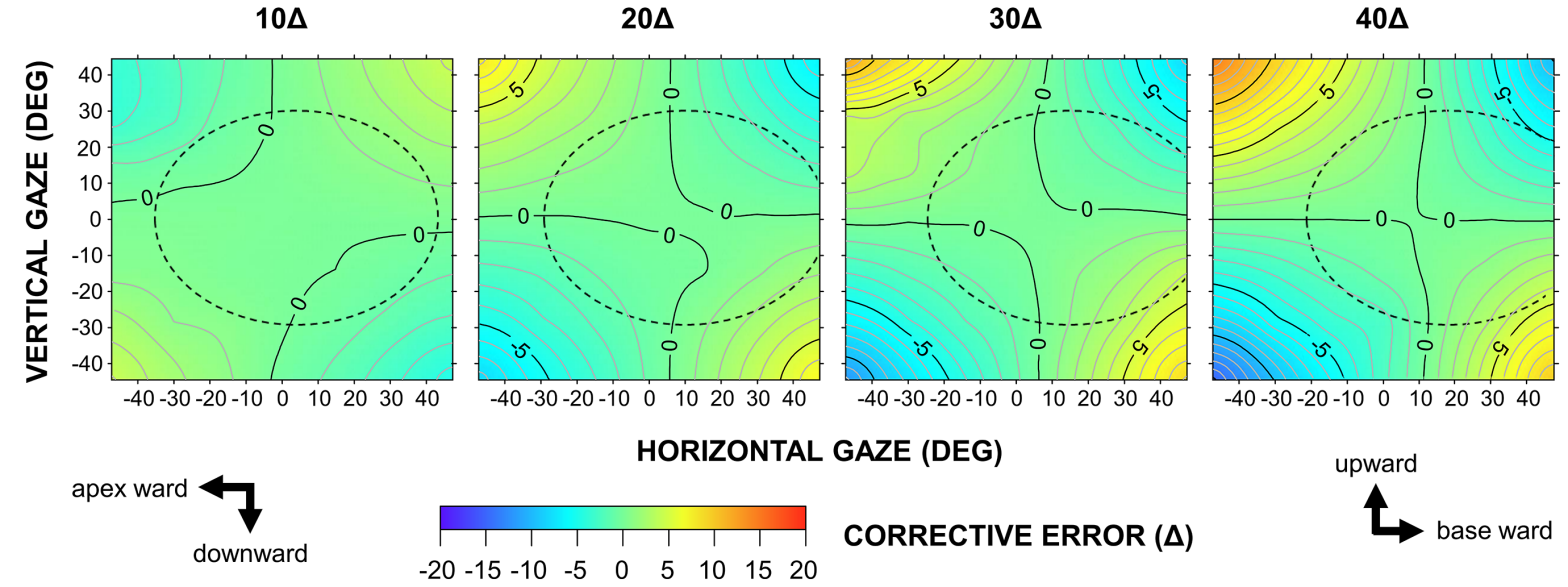
Vertical corrective errors, measured perpendicular to the principal section, for 10Δ to 40Δ Fresnel prisms in the OPS configuration.

Vertical deflection, which represents the corrective error under monocular application, is also presented as contour maps (Figs. 7, 8). Polarity was generally preserved along the diagonals of the gaze field, with the magnitude increasing from the primary to the tertiary gaze positions. As prism power increased from 10Δ to 40Δ, these error gradients became progressively steeper.

## Discussion

In the present study, Fresnel prisms produced nonlinear, gaze-dependent corrective errors in the secondary and tertiary gaze positions, even when they fully corrected the strabismic deviation in the primary position. The magnitude of these errors varied in a complex manner depending on gaze direction, prism power, and the method of application (EPS vs. OPS). In the conventional EPS configuration, prisms exceeding 20Δ induced large corrective errors, characterized by a steep gradient of overcorrection toward the apex. In addition, this bench-top experiment showed that rotating these prisms beyond 20° resulted in the disappearance of the projected spot. This phenomenon may be attributed to internal multiple reflections, total internal reflection, or occlusion by the sidewalls of the prism^14^ and may manifest clinically as the so-called apical scotoma.^2^^,^^14^ In the OPS configuration, the gradient was reversed along the horizontal axis and became shallower. These findings are consistent with Snell's law. For example, the theoretical curves for 30Δ in Figure 3 show the minimum deviation angle of 20° (toward the base) in the EPS configuration, compared to –6° in the OPS configuration. Because the curves followed a concave-down parabolic trajectory, the steepest gradient occurred on the apex side in the EPS configuration.

Interestingly, a vertical gaze shift while maintaining a constant horizontal gaze angle not only amplified the horizontal corrective effect but also generated a deflection perpendicular to the principal section of the prism. Geometrical optics provides a rationale for this finding. Briefly, an incident ray entering the prism at an oblique angle relative to the principal section traverses a tilted, triangular cross-section, the apex angle of which exceeds that of the principal section. This geometry amplifies the prism deflection along the principal section. At the rear (exit) surface of the prism, the plane of incidence rotates, generating a secondary deflection perpendicular to the principal section. However, within the typical spectacle lens area, vertical corrective errors were within 3Δ (Figs. 7, 8), suggesting that most were compensated for by fusional vergence (amplitude, 2Δ–3Δ).^18^ Horizontal trapezoidal image distortion is a well-recognized phenomenon when viewing through prisms and is defined relative to the nodal point of the eye.^2^^,^^19^ In contrast, corrective errors are referenced to the center of rotation of the eye; thus, the two phenomena share a common mechanism but differ quantitatively.

The above findings have important clinical implications by clarifying the optical behavior of Fresnel prisms. Clinicians should recognize that Fresnel prisms do not provide uniform correction of strabismic deviations across the gaze field. In particular, high-power prisms (>20Δ) can induce large corrective errors in the secondary and tertiary gaze positions that may be difficult to fuse. In adults, Fresnel prisms are commonly prescribed monocularly in the EPS configuration.^2^ When gaze is maintained in the primary position, the prism-wearing eye is oriented toward the apex, where the corrective effect exhibits the steepest gradient (Fig. 5). This configuration may adversely affect spectacle comfort. Accordingly, employing the OPS configuration with a calibrated prism may improve therapeutic outcomes by reducing overcorrection on the apex side. Fresnel prisms attached to the anterior (convex) surface of spectacle lenses have been assumed to detach easily. In our practice, they remain reasonably stable, likely due to the flatter base curves achieved by modern aspheric lens designs. Prism power may also be split between the two eyes.^2^^,^^13^ This approach halves the required prism power per eye, eliminates left–right gradient asymmetry, and can partially cancel the unintended vertical component between the two eyes. Nevertheless, overcorrection in the lateral gaze remains evident, as demonstrated by the 20Δ plot in Figure 3. The horizontal oculomotor range in humans is approximately ±55°. However, natural visual orienting behavior typically utilizes only about half of this range,^20^ and gaze shifts exceeding ±20° from the primary position are compensated for by natural head–eye coordination.^21^^,^^22^ Accordingly, patients using a Fresnel prism may be encouraged to use the central region of their spectacle lenses and adopt the “head mover” strategy.

The unique optical properties of Fresnel prisms may be strategically leveraged to improve therapeutic outcomes. For example, in abducens nerve palsy, eso-deviation increases in the paretic side gaze (attempted abduction). Applying a base-out prism to the paretic eye in the OPS configuration, rather than the conventional EPS configuration, may enhance the corrective effect during abduction (Fig. 3; open circles, gaze toward the base) and expand the field of binocular single vision. Contour maps may also be applied to vertical prisms by interchanging the *x*- and *y*-axes. In patients with hypotropia associated with thyroid ophthalmopathy involving inferior rectus restriction, applying a base-up prism to the affected eye in the OPS configuration may similarly enhance the corrective effect during supraduction (Fig. 3; open circles, gaze toward the base). Conversely, the EPS configuration may provide some benefit to patients with high accommodative convergence/accommodation (AC/A) esotropia because the corrective effect of the base-out prism increases steeply at near viewing distances (Fig. 3; filled circles, gaze toward the apex).

The benefits of PAT remain controversial.^2^^–^^12^ Although PAT is believed to serve as a preoperative simulation,^2^^–^^9^ our contour-mapping results indicate that Fresnel prisms are not functionally equivalent to muscle surgery, precluding their use as substitutes. In a monkey model, Oohira and Zee^23^ demonstrated that prismatic or optical manipulations induced orbital-position–dependent, disconjugate oculomotor adaptation, with prominent horizontal components that persisted under monocular viewing. Furthermore, in experiments in healthy subjects, Schor and Maxwell^24^^,^^25^ reported that instructing subjects to alternate fixation between two targets placed in the secondary or tertiary positions, each associated with binocular disparities of opposing vertical polarity, for 30 to 40 minutes altered ocular alignment across all gaze positions. This effect was initially global but later became gaze dependent, forming a consistent gradient along a particular meridian. Taken together, in patients with potentially intact binocular function, such as those with intermittent exotropia or acquired esotropia, PAT may not always reveal the maximum deviation. Instead, it may capture vergence adaptation to gaze-dependent corrective errors introduced by the Fresnel prism. Our bench-top measurements serve only as a hypothesis-generating framework and warrant further clinical validation.

This perspective aligns with the clinical observation that prism responders who can “eat up” a given prism power, reflecting sufficient vergence adaptability, generally exhibit better long-term postoperative outcomes.^11^^,^^26^ In contrast, a high proportion of elderly patients reportedly cannot tolerate the routine use of Fresnel prisms, despite achieving binocular single vision in the primary position.^2^^,^^27^ Optical drawbacks of Fresnel prisms, including chromatic aberration, image distortion, reflections, scattering, and diffraction, have been shown to limit patient tolerance and the overall clinical acceptance of prism-based treatment.^2^^,^^14^^,^^27^^,^^28^ Age-related decline in slow vergence adaptation^29^^,^^30^ may result in insufficient compensation for the gaze-dependent corrective errors associated with Fresnel prisms.

Several limitations of this study should be acknowledged. Our geometric model assumed a fixed-lens curvature radius of 86 mm; however, this parameter varies with lens design and refractive power. We also did not consider individual differences in lens positioning or in the center of rotation of the eye, which may have introduced deviations into the estimation of corrective errors. Furthermore, the area of apical scotoma depicted in the contour maps may be overestimated because measurements were obtained at discrete 10° intervals. In addition, we did not adopt a coordinate system to describe gaze positions for simplicity. Finally, lot-to-lot variability of the prisms was not assessed.

## Conclusions

Fresnel prisms induced nonlinear, gaze-dependent corrective errors in the secondary and tertiary gaze positions, even when fully correcting strabismic deviations in the primary position. The magnitude and spatial distribution of these errors depended on gaze position, prism power, and, notably, the method of application (EPS vs. OPS). These optical properties must be carefully considered to optimize therapeutic outcomes and ensure diagnostic accuracy.
